# Reducing Time to First Contact in a Community Older Adults Psychiatry Service

**DOI:** 10.1192/bjo.2026.11496

**Published:** 2026-06-30

**Authors:** Charlemagne Tai Ming Jake, Shanmathi Mahesh, Anthony Flynn, Dipanshu Kakkar, Maja Elia

**Affiliations:** North London Mental Health Partnership, London, United Kingdom

## Abstract

**Aims:**

National standards require that adults start to receive help within four weeks of referral, which may include advice, support, or an initial assessment. Nevertheless, meeting this standard has been increasingly challenging for the Barnet West Community Mental Health Team due to rising referral volumes and clinical complexity. This project initially aimed to increase the proportion of referrals receiving first contact within 28 days by ≥10% over 1 year. As cycle 1 surpassed this target, cycle 2 aimed for a further ≥20% over 6 months.

**Methods:**

Stakeholder consultation suggested that establishing a non-clinical Referral Coordination team could improve access without increasing clinician workload. The team would provide proactive early telephone contact to gather information, assess risk, and escalate or relay clinician advice where required.

In cycle 1, Foundation Year doctors piloted the Referral Coordination role. To minimise impact on their training, they contacted only incomplete or high-risk referrals.

Following demonstrated safety and benefit, cycle 2 employed 2 non-clinical workers to form a dedicated Referral Coordination team. They contacted all new referrals and reviewed outcomes at weekly multidisciplinary meetings.

The primary outcome measure was the proportion of referrals receiving first contact within 28 days of referral. Patient satisfaction with waiting times was measured as a secondary outcome via telephone survey. Due to high referral volumes, outcome measures were collected for a random sample of 50 patients per cycle.

**Results:**

Baseline data showed that only 24% of referrals were contacted within 28 days. Median time to initial contact was 51.5 days (range 307, IQR 53), and 34% of referrals reported satisfaction with waiting times.

In cycle 1, 44% of referrals were contacted within 28 days, with a reduced median wait of 31.5 days (range 284, IQR 73); satisfaction increased to 48%. In cycle 2, 96% were contacted within 28 days, with a median wait of 5 days (range 41, IQR 4); satisfaction was reported by 44%. Notably, only 21% reported dissatisfaction whilst 15% abstained, stating they had not wished to be referred.

**Conclusion:**

The Referral Coordination team was successful in achieving near-universal timely contact. It also acted as an early warning system for clinical risk and reduced competing demands on doctors. Patient satisfaction did not correspond directly with access, suggesting influence from contextual factors such as patient expectations. Future work mayexplore additional drivers of patient experience to ensure service changes deliver meaningful benefit for patients and carers.

